# Supplementary material for: Defining the Role of Isoeugenol from Ocimum tenuiflorum against Diabetes Mellitus-Linked Alzheimer’s Disease through Network Pharmacology and Computational Methods
Source: Molecules. 2022 Apr 7;27(8):2398. doi: 10.3390/molecules27082398 (PMC9025232; doi:10.3390/molecules27082398)
Supplement: Supplementary file 1 [file molecules-27-02398-s001.zip › molecules-1648289-supplementary.pdf]

Supplementary material

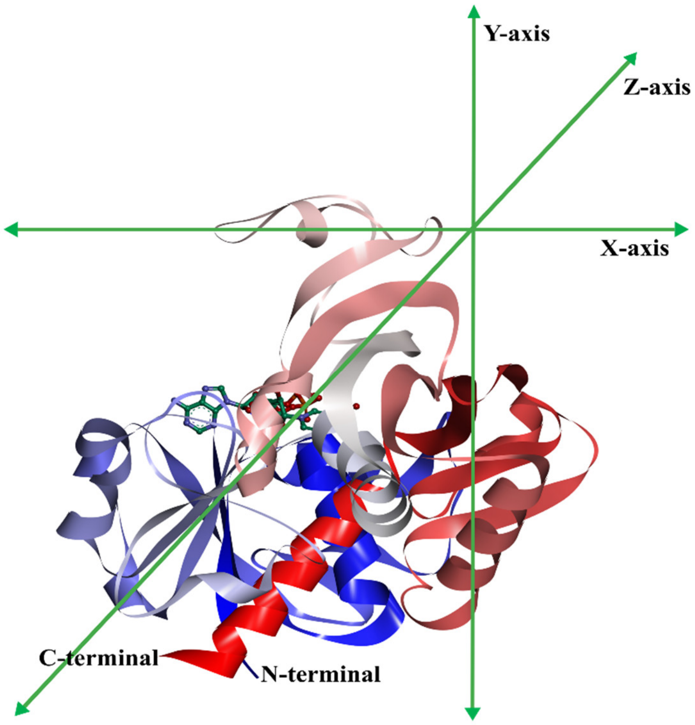

A

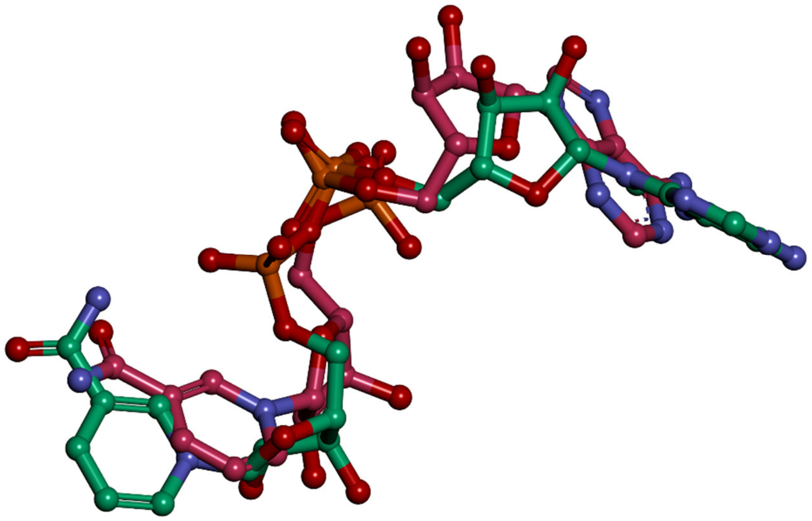

B

Figure S1: A) Structural details of protein GAPDH (PDB ID: 1U8F) bound to its co-crystallized NAD+ cofactor. N-terminal of the protein is highlighted in blue, whereas the red terminal is highlighted in red. B) Result from the validation of the docking protocol: Crystallized and bound NAD+ (green) complexed with re-docked NAD+ (red) with an RMSD of 2.10 Å.

Residues bound with NAD^+	Compounds					
	Isoeugenol			Myrcene		
ASN 9	0	0	0	0	0	0
GLY 10	0	0	0	0	0	0
GLY 12	1	0	0	0	0	0
ARG 13	1	2	0	0	0	0
ILE 14	0	2	0	0	0	2
ASP 35	0	0	0	0	0	0
PRO 36	0	0	0	0	0	0
PHE 37	0	0	0	0	0	0
ARG 80	0	0	0	0	0	0
SER 98	1	0	0	0	0	0
THR 99	0	0	0	0	0	0
GLY 100	0	0	0	0	0	0
SER 122	0	0	0	0	0	0
CYS 152	0	0	0	0	0	0
ASN 316	0	0	0	0	0	0
TYR 320	0	0	0	0	0	0
Occupancy (%)	43.75			12.5		

	Hydrogen bonds
	Pi-alkyl bonds
	Alkyl bonds

Figure S2: Protein-ligand fingerprinting of isoeugenol and myrcene docked with GAPDH.
